# Aggregating and analysing clinical trials data from multiple public registers using R package ctrdata

**DOI:** 10.1017/rsm.2025.10061

**Published:** 2025-12-04

**Authors:** Ralf Herold

**Affiliations:** Regulatory Science and Innovation Taskforce, European Medicines Agency, Netherlands

**Keywords:** Clinical trials, study protocol, trial results, trial registers, meta-analysis, R package

## Abstract

The ctrdata package has been created to boost the use of data available in public registers of clinical trials. It enables user-friendly, reproducible workflows to identify trials of interest, download protocol- and results-related data, and conduct sophisticated analyses, across multiple registers and trials. ctrdata works in the widely used R environment, and its databases can be used with other tools. The package is open source with a permissive licence, to facilitate collaboration.

This report provides an overview of ctrdata, including its implementation, cases of interest to researchers in public health, medicines, and regulatory science, as well as potential limitations and further developments. At this time, ctrdata works with the European Union (EU) Clinical Trials Information System (CTIS), the EU Clinical Trials Register (EUCTR), the US Clinicaltrials.Gov (CTGOV), and the ISRCTN—the UK’s Clinical Study Registry. The registers are complementary in scope and scientific value, yet differences in data models, variable definitions, search parametrisations, and retrieval options hamper efficient scientific workflows, calling for a scientific-technical, programmatic solution and driving the development of ctrdata.

By employing ctrdata to comprehensively use and easily leverage trial register data, researchers can effectively address a variety of questions, gain useful insights into evolving policies and practices of drug development, and inform further clinical research. Patients and their organisations, developers, policymakers, and other interested parties can build on ctrdata to create solutions for their use cases.

## Highlights

### What is already known?

- Clinical trials are a primary approach for generating evidence on health interventions. Trials are regulated to ensure participants’ well-being, scientific relevance, and transparency.
- Registers make public rich trial data, but tools for efficiently using the data are lacking, impacting reproducible, deep information synthesis and learning.

### What is new?

- ctrdata, an open-source R package, enables using all publicly available data and documents from four registers (the EU Clinical Trials Information System [CTIS], the EU Clinical Trials Register [EUCTR], the US Clinicaltrials.Gov [CTGOV], and ISRCTN—the UK’s Clinical Study Registry). It supports research steps from identifying and storing trials of interest, deduplication, scrutinising structure, extracting fields, to analysing user- or pre-defined scientific and operational concepts.

### Potential impact for RSM readers

- With ctrdata, researchers can easily implement a programmatic workflow to investigate trials in depth. ctrdata keeps pace with register changes and user requirements. Its databases can be used with any system.
- Beyond drug development, ctrdata is relevant for patient access, methodology research, health policy, and outcomes research.

## 1. Introduction

Clinical research should be well informed by evolving experience, for which public registers have become a transparent source and comprehensive reference. However, it is increasingly difficult to scrutinise trials and understand their design, conduct, and results, because their number and complexity is growing fast.^1^

Registers are an important means by which sponsors and regulators increase the transparency on clinical trials^2^ for the benefit of patients, health professionals, researchers, and developers, whether from the academic or for-profit sector. Registers offer more or less user-friendly web interfaces, for manual finding and reviewing of specific trials of interest. Yet, surveys of the general public in European countries led Parsons et al. to conclude that ‘public interest in medicines R&D was greater than public knowledge, which suggests that attempts to increase public knowledge will be welcomed’.^3^ With such attempts, researchers, health professionals, and patients ‘can identify knowledge gaps that need to be filled with new trials’.^4^

There is, however, a lack of tools that enable an efficient scientific-technical or programmatic approach to analyse register data from individual and sets of trials. A report on the underutilisation of registers noted the extra effort and time required for manual screening of trials.^5^ Linkage and synthesis of trial data across registers is hampered by differences between the registers’ data models, that is, by different variable structures and value lists used for corresponding information concepts. While the WHO International Clinical Trials Registry Platform (ICTRP) could be seen as an example of data linkage, it covers only a limited subset (24 items in the ‘WHO Trial Registration Data Set’), importantly without any results-related data. A screening of PubMed in July 2025 for meta-analyses or systematic reviews from the past ten years and the term ‘Clinicaltrials.Gov’, ‘ICTRP’, or ‘EudraCT’ yielded around 10500, 1800, and 60 results, respectively, with only some 35 results using both the US Clinicaltrials.Gov (CTGOV) and the EU Clinical Trials Register (EUCTR).^6^ In the scientific literature, there seems a scarcity of reports of EUCTR and cross-register analyses. The complexity and continual evolution of register data are part of the technical challenges with data fragmentation across health data systems and with advancing data linkage between such systems.

The package ctrdata is a recent yet mature tool that facilitates accessing all public protocol- and result-related information on clinical trials in registers.^7^ The functionality covers identifying, querying, downloading, aggregating, and analysing data across registers, including historical versions of trials and trial-related documents, as far as publicly available in registers. The package ctrdata is available as open source with a permissive licence, and collaborations are welcome to increase its usefulness as a tool. Package ctrdata works with the EUCTR, the EU Clinical Trials Information System (CTIS), the CTGOV, and ISRCTN—the UK’s Clinical Study Registry.

The intention with ctrdata is to maximise the usefulness of trial registers for increasing public knowledge, for participation in research, for informing on health interventions, for decision-making of patients and professionals, and for efficient future clinical research.

Creating package ctrdata was also motivated by questions in regulatory science that led to research activities within the European Medicines Agency (EMA), such as on the relation of juvenile animal studies and clinical trials in children^8^ and on trends in clinic research during the COVID-19 pandemic .^9^

The leading idea is that ctrdata encompasses and abstracts register-specific parts of a workflow, on top of which users can build their generic trial workflow parts and applications.

This is the first report on ctrdata, and it covers the technical background, several use cases of likely interest to researchers in public health, medicines, and regulatory science, and a discussion of potential limitations and future developments.

## 2. Implementation

Package ctrdata provides a system for clinical trials data that includes loading from registers, storing and extracting for analysis and re-use.

**Figure 1:**
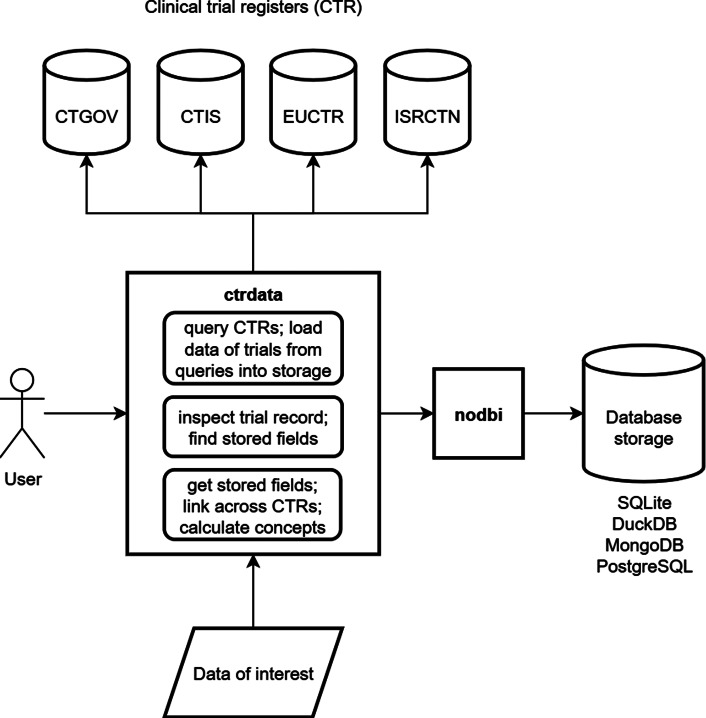
Overview on using R package ctrdata. The arrow means ‘makes use of’. The user can execute functions in package ctrdata that query clinical trial registers (CTRs) and load data of trials of interest. Such functions include ‘ctrGenerateQueries()’, ‘ctrLoadQueryIntoDb()’, and ‘ctrFindActiveSubstanceSynonyms()’. These functions make use of the application programming interfaces (APIs) and web interfaces of four CTRs. The user can inspect trials with ctrdata function ‘ctrShowOneTrial()’ and select fields with ‘dbFindFields()’. Package ctrdata uses package nodbi for storing clinical trial data in SQLite, DuckDB, MongoDB, or PostgreSQL. Data of interest are generated using ctrdata functions ‘dbGetFieldsIntoDf()’ (which extracts data from the database, combines data from different registers into a data set, and calculates concepts across trials), ‘dfTrials2Long()’, and ‘dfName2Value()’ (which reshape a data set and select nested fields based on field identifiers).

An overview on the main components that ctrdata provides and that ctrdata uses is in Figure 1.

The following sections discuss main ctrdata components.

### 2.1. Registers

When the development of ctrdata was started in 2015, making public information on registered clinical trials had been required by US legislation since fifteen years,^10^ by medical journal editors for ten years,^11^ and by legislation in the European Union (EU) for five years.^2^

At that time, CTGOV provided an API,^12^ and both CTGOV and EUCTR used XML schemas (Extensible Markup Language)^13^ for data models, published them for information of data providers, and continually updated them to meet business requirements and changes in the legislative frameworks.^14^ ^,^ ^15^

Package ctrdata supported these two registers since 2015. The ISRCTN is supported since 2021, when it was started to be used for statutory purposes in the UK; it provides an API and XML data.^16^

Since March 2023, ctrdata supports the EU CTIS, both for data made public before and after its relaunch in mid-2024.^17^ CTIS is to be used for new clinical trials since February 2022. Data from CTIS are derived in JSON format (JavaScript Object Notation^18^) from its public API that feeds the register’s web interface.

The current main characteristics of registers are summarised in Table 1.

**Table 1:** Overview of registers supported by ctrdata (numbers rounded to three significant digits; XML, extensible markup language; JSON, JavaScript Object Notation; ‘other types of clinical studies’ refers to studies of medical devices, behavioural and other health interventions, observational, non-interventional, and other studies)

Information as of end July 2025	EU Clinical Trials Register	ClinicalTrials.gov	ISRCTN registry	EU Clinical Trials Information system
Protocol-related data published	Text in special format	JSON (since 2024)	XML	JSON
Results-related data published	XML and / or documents, variably	JSON (since 2024)	No structured data, but links to publications	No structured data, but PDF document(s)
Application programming interface (API)	No	Yes	Yes	Limited (no documentation)
Documents available for download	Yes, typically result reports or publications	Yes (accepted since 2017), protocol, statistical, analysis plan, and (since 2024) informed consent forms	No	Yes, broad range such as consent forms, protocol, statistical analysis plan
Trial identifier format	1234–123456–12 (EudraCT number), 1234–123456–12-AB (country version)	NCT12345678	12345678	1234–123456–12–12
URL in user’s browser identifies trial search or individual trial	Yes	Yes	Yes	No, but ctrdata includes a tool to provide query URLs for CTIS (see Section 3.1)
Register identifier in ctrdata	EUCTR	CTGOV2 (since 2024)	ISRCTN	CTIS
Number of trial records	44400	546000	26700	9620
Number of trial records with results (portion of all records)	25300 (57%)	73000 (13%)	14500 (54%)	236 (2%)
Interventional trials with medicinal products	Yes, exclusively	Yes (estimated 40%), together with other types of clinical studies, see https://clinicaltrials.gov/about-site/about-ctg#q3	Yes (estimated 12%), together with other types of clinical studies, see https://www.isrctn.com/page/faqs	Yes, exclusively
Sources on statutory aspects of registration and publication	https://www.clinicaltrialsregister.eu/about.html; in particular, no data are made public on most phase 1 trials	https://clinicaltrials.gov/policy; see, for example, checklist for definition of ‘Applicable clinical trial’ (ACT)	https://www.isrctn.com/page/resources; in particular, automatic registration of all clinical trials by the UK ‘Health Research Authority (HRA)’ since 2022	https://euclinicaltrials.eu/guidance-and-q-as/; in particular, ‘Revised CTIS Transparency Rules’, some data on all trials to be made public at time of regulatory decision on the clinical trial authorisation

### 2.2. Principles

Several principles evolved when developing package ctrdata.

All details are downloaded for clinical trials of interest, because only a complete public record is an accurate representation of the trial. Since the registers differ in scope, legal, and regulatory purposes, their content is complementary and ctrdata thus should work with several and an increasing number of registers.

Before analyses, trial information should first be downloaded and stored in a database, because the set of trials of interest is often a union set from different search queries and possibly different registers, and because offline availability of data for analysis is useful.

The data models that are implicit in data as retrieved from the different registers are retained by ctrdata, because differences between the data structure and value sets of different registers can well be handled at the time of analysis, and because any mapping to a putative target data model would be a goal suitable for an international harmonisation organisation.

Against this technical background and principles, ctrdata was implemented in the R environment,^19^ which has a broad user base and has an extensive support for structured data, network operations, dependency management, and quality assurance.

At the same time, the users of ctrdata should be provided with functions that are simple and cover all relevant steps, without duplicating functions in R or one of its many extension packages. Main functions in ctrdata are listed in Table 2 in the order of a potential workflow, together with the number of the use case in Section 3 that exemplifies the function.

**Table 2:** Main functions in ctrdata in order of a potential workflow

Function name	Purpose	Use case
ctrGenerateQueries()	From simple user parameters, generate queries for each register to find trials of interest	1, 8
ctrLoadQueryIntoDb()	Retrieve (download) or update, and annotate, information on trials from a register and store in a collection in a database	2, 8, 9
dbQueryHistory()	Show the history of queries from which trial data was loaded into the database collection	2, 7
ctrShowOneTrial()	Show full structure and all data of a trial, interactively select fields of interest for ‘dbGetFieldsIntoDf()’	2
dbFindFields()	Find names of data fields in the collection	2
dbGetFieldsIntoDf()	Create a data frame (or tibble) from trial records in the database with the specified fields. Returns specified fields and specified clinical trial concepts calculated across registers, see Table 4	2, 3, 4, 5, 6, 7, 8
dfMergeVariablesRelevel()	Merge selected, related variables in a data frame (or tibble), keeping their type where possible, and optionally re-level factors to new categories	4
dfTrials2Long()	Transform the data frame from ‘dbGetFieldsIntoDf()’ into a long name-value data frame, including deeply nested fields	10
dfName2Value()	From a long name-value data frame, extract values for data fields of interest (e.g. endpoints)	10

*Note:* A full list of functions is part of the documentation website at https://rfhb.github.io/ctrdata/.

### 2.3. Analysis concepts

Package ctrdata includes functions that implement specific analysis concepts (Table 3). Concepts of clinical trials, such as their start date or their number of arms/groups with different investigational medicines, require analysing several fields against various criteria. However, the structure and the value sets of data fields differ between the registers.

**Table 3:** Overview on clinical trial concept functions implemented in ctrdata

Function name	Value types of column(s) returned by function	Question addressed by function	Use case
f.assignmentType()	Factor (‘R’ or ‘NR’)	Was the assignment to treatment based on randomisation or not? Since ISRCTN does not have a specific field, a text pattern is used for calculation.	
f.controlType()	Factor (‘none’, ‘no-treatment’, ‘placebo’, ‘active’, ‘placebo+active’, or ‘other’)	Which type of internal or concurrent control(s) is/are used in the trial? For ISRCTN, a text pattern is used for calculation.	6
f.externalLinks()	Character	Which links to publications and other external references are referenced from a study record?	
f.hasResults()	Logical	Have any results been recorded for the study record, such as structured data, reports, or publications?	
f.isMedIntervTrial()	Logical	Is the trial interventional and does it have one or more medicines (drugs or biological) as investigational (experimental) intervention? Calculated irrespective of medicine authorisation.	
f.isUniqueTrial()	Logical	Is the trial record unique in the data frame of trials? This function is based on ‘dbFindIdsUniqueTrials()’ with its default parameters.	3
f.likelyPlatformTrial()	Logical, list of character, list of character	Is the trial likely a (research) platform trial, and what are related trials? Provides new columns with a logical indicator, a list of likely related trials, and a list of possibly related trials. For the logical indicator, a temporary approximation was defined at this time as follows. The study record is likely a platform trial if at least one of the following criteria is true: a) The study has ‘platform’, ‘basket’, ‘umbrella’, ‘multi.?arm’, ‘multi.?stage’, or ‘master protocol’ in its title or description (for ISRCTN, this is the only criterion) b) The study has more than two active arms with different investigational medicines, as calculated with f.numTestArmsSubstances() c) The study has more than two periods after excluding safety run-in, screening, enrolling, extension, and follow-up periods (for CTGOV and CTGOV2, this criterion requires results-related data) The list ‘.likelyRelatedTrials’ is based on other identifiers provided in the trial record, e.g. ‘associatedClinicalTrials’ in CTIS; identifiers are included in the list whether or not the trial with that identifier is in the database collection. The list ‘.maybeRelatedTrials’ is at this time based on the similarity of parts of the trial title that may be acronyms or codes of a master protocol; identifiers can be included in the list only from trials that are in the database collection.	
f.numSites()	Integer	How many sites does the trial have?	
f.numTestArmsSubstances()	Integer	How many arms or groups have different investigational medicines, after excluding non-active comparator, auxiliary and placebo arms / medicines? This cannot be calculated for ISRCTN or for phase 1 trials. The temporary approximation uses string similarity to identify different active substances with 0.8 optimal string alignment metric threshold.	
f.primaryEndpointDescription()	List of character	String containing protocol definition, details, and time frames, concatenated with ‘==’ for each primary endpoint	5
f.primaryEndpointResults()	Number, character, integer	Provides news columns with the statistical testing p-value and method as well as the number of subjects included in the test, for the first primary endpoint only	6
f.resultsDate()	Date	The planned or achieved date of results availability	
f.startDate()	Date	The planned, authorised, or documented date of start of recruitment	8
f.sampleSize()	Integer	The planned or achieved number of subjects or participants recruited	3
f.sponsorType()	Factor (‘not for profit’, ‘for profit’, ‘mixed’, or ‘other’)	Simplified class of sponsor(s) of the study	5
f.statusRecruitment()	Factor (‘ongoing’, ‘completed’, ‘ended early’, or ‘other’)	Simplified status; ‘ongoing’ includes temporarily halted, ‘ended early’ includes terminated or ended prematurely, and ‘other’ includes planned, stopped, withdrawn	3
f.trialObjectives()	String	Identifies with letters those objectives that could be identified by text fragments, e.g. ‘E S PD D’, with ‘E’ (efficacy), ‘S’ (safety), ‘D’ (dose-finding)	5
f.trialPhase()	Ordered factor (‘phase 1’, ‘phase 1+2’, etc.)	Phase(s) of medicine development with which a trial is associated	3, 5, 7
f.trialPopulation()	Factor, string, string	Provides new columns for age group(s) (e.g. ‘P’ for paediatric participants, ‘A’ for adults, ‘E’ for participants older than 65 years, or ‘P+A’), the inclusion and exclusion criteria texts	
f.trialTitle()	String	Full or scientific title of the study	Annex

*Note:* The refinement of some of the concepts is informed by ongoing research and use cases.

To address this situation, 20 trial concepts, pre-defined in ctrdata, are offered to simplify and accelerate a user’s analysis workflow, thereby increasing analysis consistency and reproducibility.

Some trial concepts can build on clear definitions and close similarities of registers; thus, concepts such as the trial phase, trial population, number of sites, and status of recruitment when loading the trial can be calculated with some confidence, yet users should note the respective help texts, which include any caveats such as that EUCTR does not have numbers of sites for non-EEA (European Economic Area) countries .

Where definitions are not closely similar, an operational definition was chosen to create trial concepts of interest; an example is the sponsor type at the level of the trial, for which a new value of ‘mixed’ is calculated if a trial’s sponsors include commercial and non-commercial entities.

Other trial concepts reflect the author’s proposals for temporary approximations of less well-defined concepts, such as trial objectives (e.g. dose-finding, pharmacodynamics) and ‘f.likelyPlatformTrial()’, with the function name flagging the uncertainties.

The trial concepts in ctrdata (all described in Table 3) have not been validated with any formal approach but have been checked for plausibility and against common sense expectations. Where possible, the implementation of a trial concept is based on documented current understanding, on public data models, or on scientific papers, as relevant. Users are invited to note the help texts of the concepts, which mention the register fields and any caveats, to review the function logic in its code (as in Section 3.3), and to raise an issue or to contribute improving any trial concept in the public repository of ctrdata (see data availability statement).

### 2.4. Storage

For storage of trial data, a document-centric approach was chosen because all data on a particular trial represent a self-standing document, where documents can differ in structure and do not require to pre-specify a schema.

The R package nodbi is used as a connector to document-centric databases and was extended to work, in addition to MongoDB, with PostgreSQL, RSQLite, and DuckDB as backend.^20^ The latter are SQL databases but have functions for handling JSON which are abstracted by nodbi so that all four backends can be used interchangeably, without further changes in R scripts. Since RSQLite and DuckDB are available for all R platforms as local databases, their use with package ctrdata is likely of general interest.

Databases created with ctrdata can be accessed with other R packages and with other languages, such as Python, Julia, or JavaScript. Furthermore, using a MongoDB server enables to execute analyses directly on the server, such as efficient aggregation pipelines as shown in one of ctrdata’s vignettes.^21^

## 3. Use cases

The ten use cases in this section are diverse examples to illustrate how research questions can be addressed with ctrdata. A general workflow is shown in the sequence of functions in Table 2. The results of the use cases are not commented or interpreted, since the intention is to exemplify just the functionality, without scientific review or discussion.

In R, package ctrdata is installed as follows:

**Figure:**
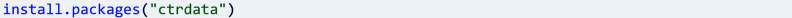

Then, the package can be loaded, here together with the package ‘dplyr’ for pretty printing and scripting:

**Figure:**
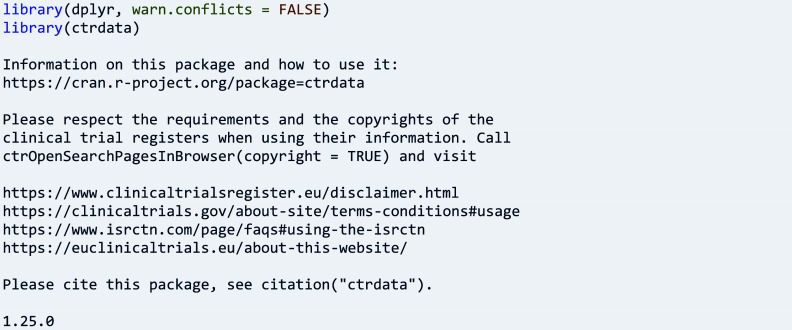

Information on CTRs is provided in package ctrdata, such as links to their documentation, reference pages, data structure, and value set descriptions:

**Figure:**
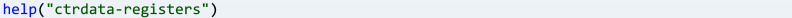

Importantly, users can open empty search and expert search pages as well as review the copyrights pages of registers for their acknowledgement before going further as follows:

**Figure:**
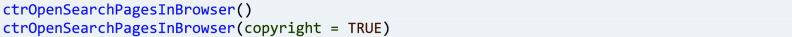

All registers (except CTIS) show in a web browser the URL that represents the user’s current trial search. This URL can be manually copied by the user and pasted as input for ctrdata to load the trial data, as exemplified below.

For convenience, a script is provided alongside package ctrdata that can be installed in the web browser, where it automatically copies register search URLs to the clipboard of the user’s device.^22^ To this end, the user would first install the Tampermonkey browser extension and then import the script located at https://raw.githubusercontent.com/rfhb/ctrdata/master/tools/ctrdataURLcopier.js. The browser extension and the script can be disabled and enabled by the user at any time.

This script is particularly useful with CTIS, where it can modify the URL as shown in the web browser to reflect the user’s parameters for searching this register. Additionally, this script can show search results in CTIS when opening URLs such as https://euclinicaltrials.eu/ctis-public/search#searchCriteria={“status”:[3,4]}; without the script, such query URLs have no effect in CTIS at this time.

### 3.1. Generate queries and count trials

Research often starts with developing a search strategy for information of interest. To facilitate searching in different CTRs, the user can provide high-level search parameters to function ‘ctrGenerateQueries()’. The parameters are translated into the different approaches of the trial registers for producing search results:

**Figure:**
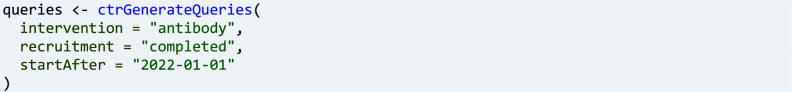

The function generates by default queries limited to interventional studies with medicines, referred to as ‘clinical trials’ throughout this article. The function parameter ‘onlyMedIntervTrials’ can be set to FALSE to remove this limitation and find all types of studies available in the register. Other interesting parameters of function ‘ctrGenerateQueries()’ are, for example, ‘countries’, ‘searchPhrase’, ‘condition’, ‘phase’, and various dates.

The function generates a (named) vector of hyperlinks specific to the registers. The links can be used to open the registers’ results pages so that the user can check and refine the queries:

**Figure:**
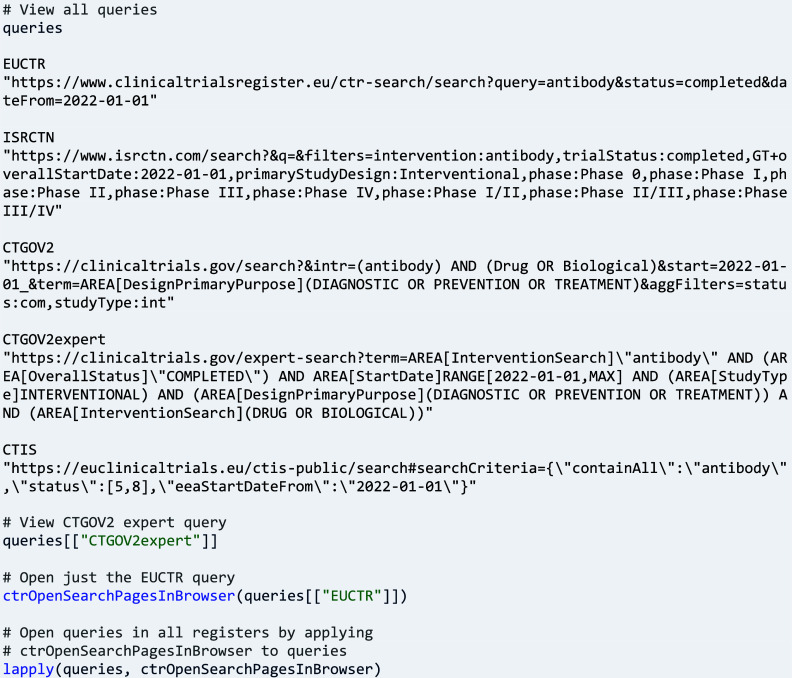

A next step can be to determine the number of trials that can be obtained with the queries. To this end, function ‘ctrLoadQueriesIntoDb()’ is executed, and this emits messages for the user’s information about the data exchange with the CTR, including the number of trials found.

**Figure:**
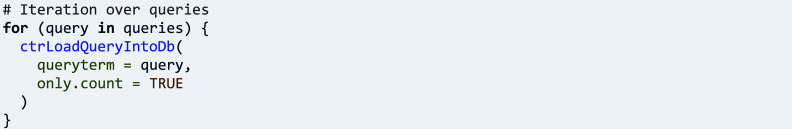

As a more advanced programming pattern, function ‘ctrLoadQueryIntoDb()’ can be applied to all queries to store return values in a list, from which the number of trials can be extracted as follows:

**Figure:**
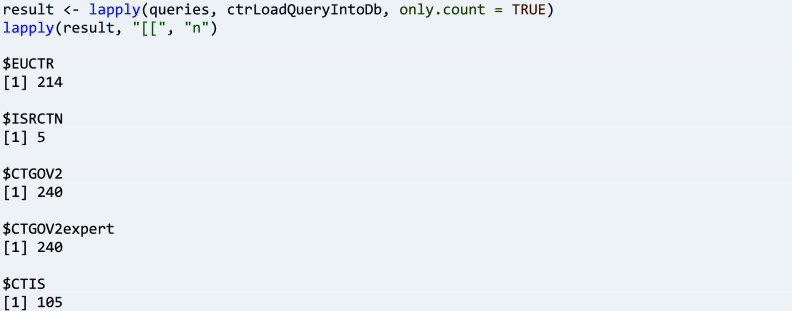

Note that the number of EUCTR records reflects the number of countries involved; it is thus a multiple of the number of trials. Also, note two types of queries are provided for CTGOV, including the register’s expert search page for interactively composing and executing more complicated and nested queries; they result in the same set of trials and thus, one query can be removed for subsequent use cases:

**Figure:**
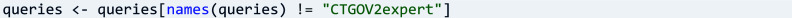

Importantly, users retain full control over queries to match their specific research interest, for example by modifying the query strings as one would modify any other string in R. Function ‘ctrGenerateQueries()’ will remain useful to get started, since searches much differ between registers.

### 3.2. Download trial data for analyses

The trials that have been identified with a search strategy have to be retrieved and downloaded, in order to refine the set of trials of interest and to analyse any of their details. One of the principles recognised by ctrdata (see Section 3.2) is that a final set of trials of interest often results from complementary queries in the same or in different registers.

First, a connection to a database is created, here SQLite (DuckDb, MongoDb, and PostgreSQL can also be used), for which the corresponding R package needs to be installed. A collection (database table) is specified to hold data of trials of interest:

**Figure:**
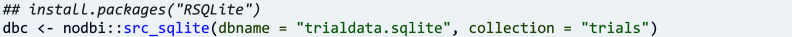

Second, the queries defined above are used to download the trial data. Function ‘ctrLoadQueryIntoDb()’ here is applied to all queries:

**Figure:**
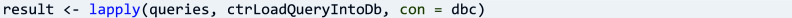

For the total of almost 1700 trial records from four registers, the downloading takes around 100s (at a maximum bandwidth of 10 MB/s; ctrdata throttles the number of requests per time period).

Any number of additional queries can be loaded into the same collection, for example:

**Figure:**
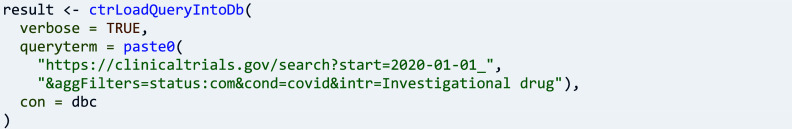

Function ‘ctrLoadQueryIntoDb()’ can also repeat a query to load trials that were updated or are new since the last time the query was loaded. Since this is a main function of package ctrdata, its full signature provides an overview of the options with which a user can tailor the data to be loaded to their needs. Some of the options are discussed in the following use cases.

**Figure:**
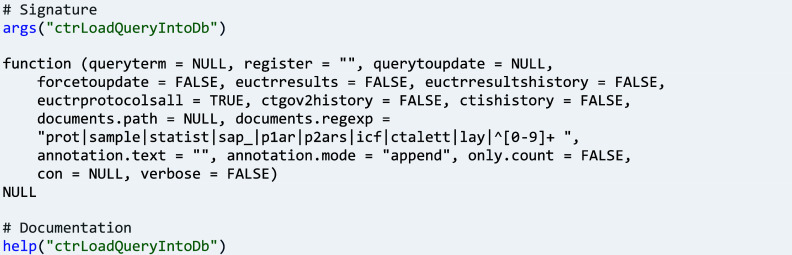

The function returns the number and identifiers of trials that were successfully loaded or failed to load, and the query that was used.

Further for documentation and reproducibility, ctrdata includes metadata in the database collection whenever ‘ctrLoadQueryIntoDb()’ is run so that users can check and re-use:

**Figure:**
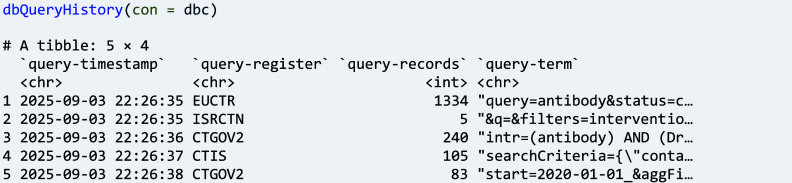

A second main function of ctrdata has the purpose to provide a user-friendly table (data frame in R) from any data in a database collection, which can then be tabulated, for example:

**Figure:**
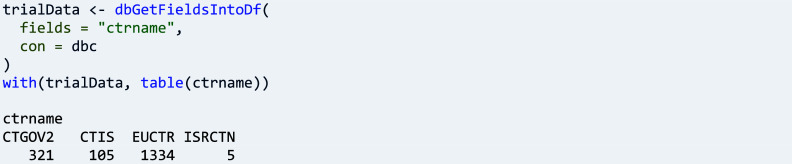

Since trial data are extensive and hierarchically structured, a user can explore their structure and value sets of individual trials with ctrdata, which provides an interactive browser widget to identify individual data fields of interest:

**Figure:**
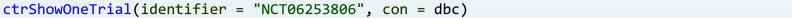

Fields of interest can also be found across a sample or all trials from different registers in a collection:

**Figure:**
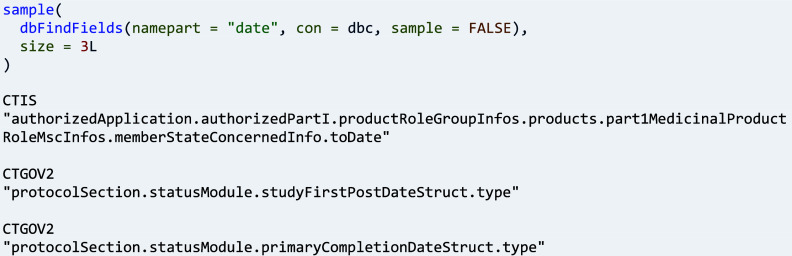

### 3.3. Analyse using pre-defined trial concepts

Beyond individual fields of interest as identified above, ctrdata comes with various trial concepts that are already implemented as functions for selecting and analysing fields from different registers.

It is seemingly simple to calculate the start date of a trial and its current recruitment status, yet it involves more than 20 fields across the registers to calculate the new columns that correspond to these two concepts for the data frame provided by ‘dbGetFieldsIntoDf()’:

**Figure:**
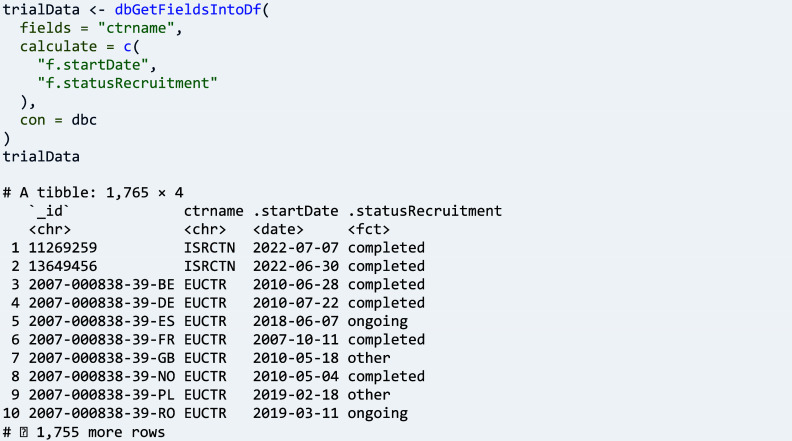

The pre-defined trial concepts much simplify a user’s workflow, and an overview on the currently 20 functions is available in Table 3 and here:

**Figure:**
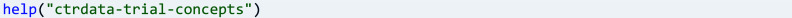

The function names indicate if a trial concept can be calculated exactly as above or can only be approximated (e.g. ‘f.likelyPlatformTrial()’). Users can inspect how a concept is calculated by calling the name of the function, for example:

**Figure:**
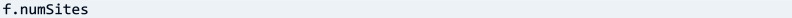

A particular trial may have been registered in more than one register, and in EUCTR one trial has one record for every participating EU Member State. Therefore, ctrdata provides the trial concept ‘f.isUniqueTrial()’, which helps to identify and select only unique trials before further analyses, to avoid double-counting:

**Figure:**
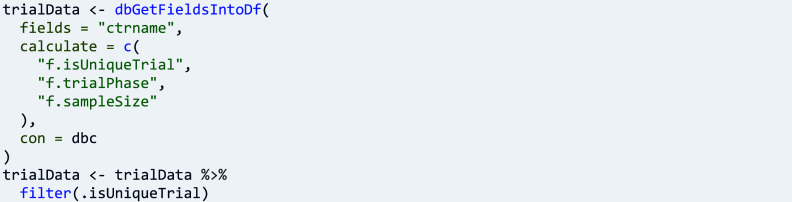

Alternatively to the above, at the time of loading trials from EUCTR, it is possible to just load a single record for any trial, by calling function ‘ctrLoadQueryIntoDb()’ with parameter ‘euctrprotocolsall’ set to FALSE. This setting can be useful when there are no questions about differences between Member States’ versions, such as dates, authorisation decisions, ethics opinions, and trial end.

**Figure 2:**
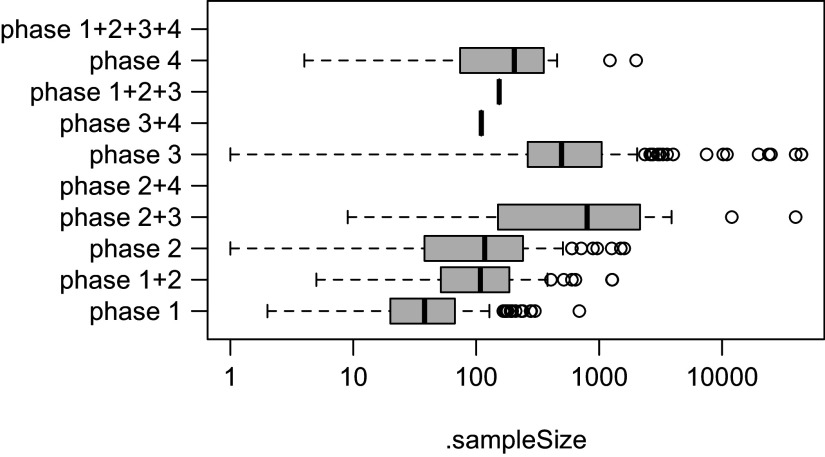
Boxplot of sample size by phase of trial.

In the example above, phases of medicine development are calculated based on values recorded in registers, and sample sizes are calculated to reflect the planned or the achieved number of participants, depending on the status of recruitment. After calculating the trial concepts, further analyses become reasonably simple, such as exploring associations (Figure 2):

**Figure:**
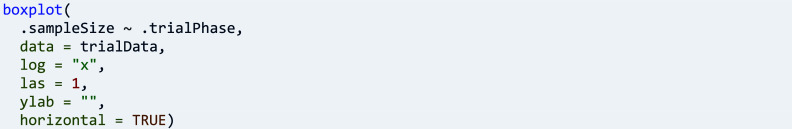

### 3.4. Merge and analyse information

Function ‘dfMergeVariablesRelevel()’ can be used for combining arbitrary fields of the same type from different registers into a new variable, here in an example for country data.

Clinical trials often span countries and even regions, in particular when conditions under study are rare, when a large number of participants is sought, or when the performance of interventions in the context of local health systems is to be analysed.^23^ All trial registers supported by ctrdata provide data on countries, and CTIS and CTGOV provide data on individual sites, including their location and contacts. The respective fields are extracted into a data frame and then are merged into a new variable, concatenated with ‘ / ’:

**Figure:**
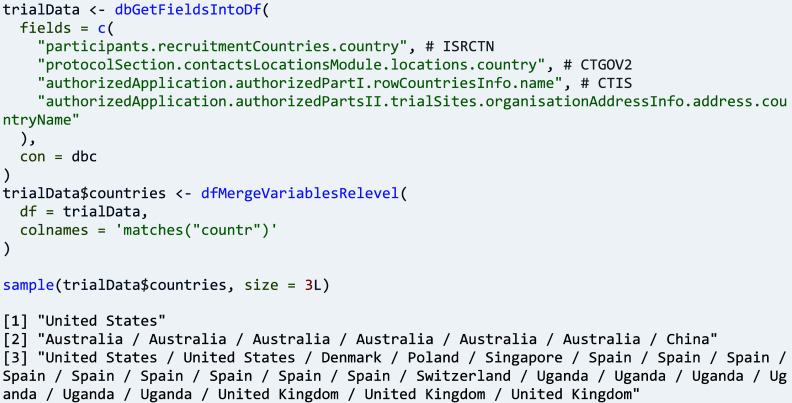

The new variable can be used for example for a cross-tabulation against all countries involved in the trials of interest, or for counting the sites per country as follows:

**Figure:**
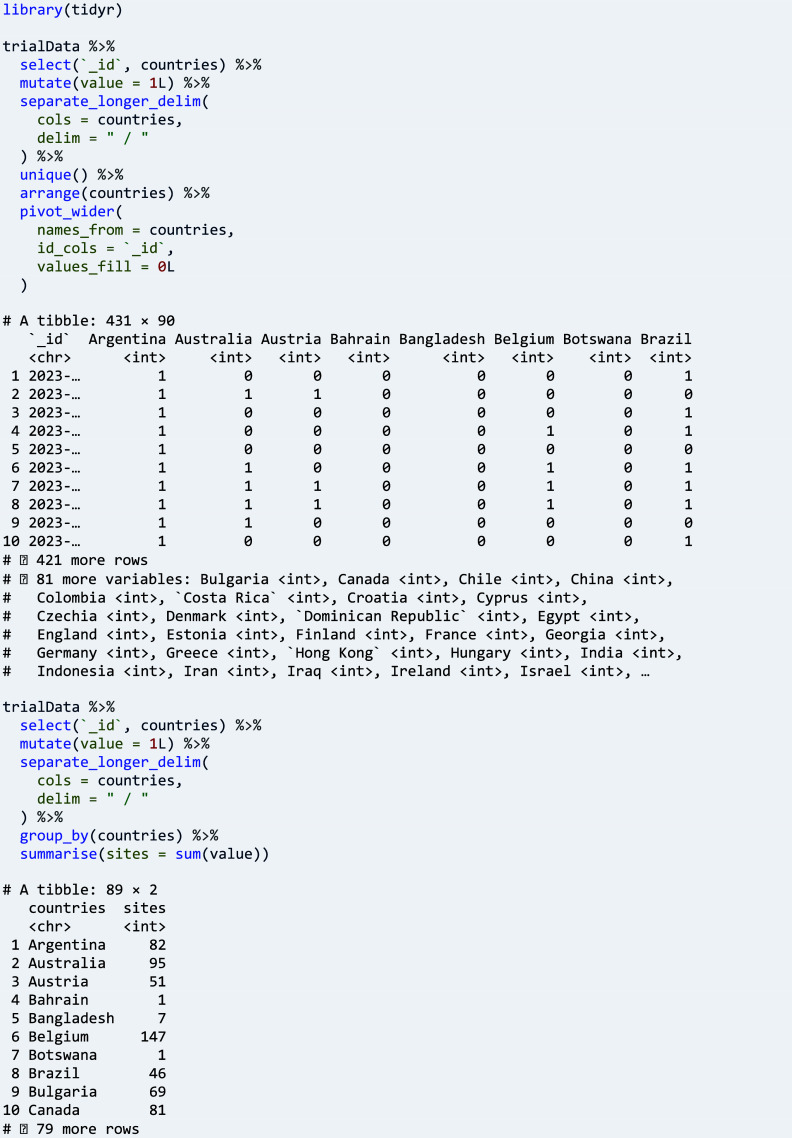

Besides analysing country data, function ‘ctrGenerateQueries()’ can be used for searching trials that are conducted in countries specified by the user.

### 3.5. Analyse text data that describe endpoints

Endpoints or outcomes investigated in clinical studies are so far represented in registers as textual descriptions, and there is no controlled terminology established globally or in any register. A particular endpoint is usually described with components covering a title or short description, its operational definition, and the time points when it is evaluated, and together they roughly correspond to the variable, one of the four estimand components.^24^ The trial concept ‘f.primaryEndpointDescription()’ can provide the outcome variable in a single string; however, text analysis methods then need to be applied.

For example, questions may concern in how far difference-from-baseline variables continue to be used. Here, for unique efficacy trials with a phase 3 label, the text analysis method is using a regular expression to determine if the primary endpoint of a trial likely corresponds to such a variable or not, cross-tabulating with the type of sponsor:

**Figure:**
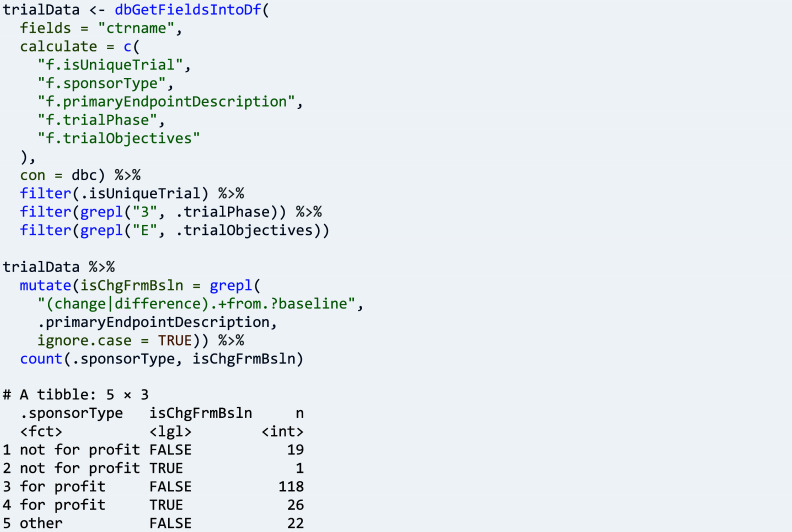

Other research questions require an abstraction or categorisation of endpoint variables, such as questions about the type of endpoint.^25^ Here, package ctrdata can be used for obtaining and pre-processing endpoint data (shown above), which then could be fed into a suitable large language model to predict the sought category:

**Figure:**
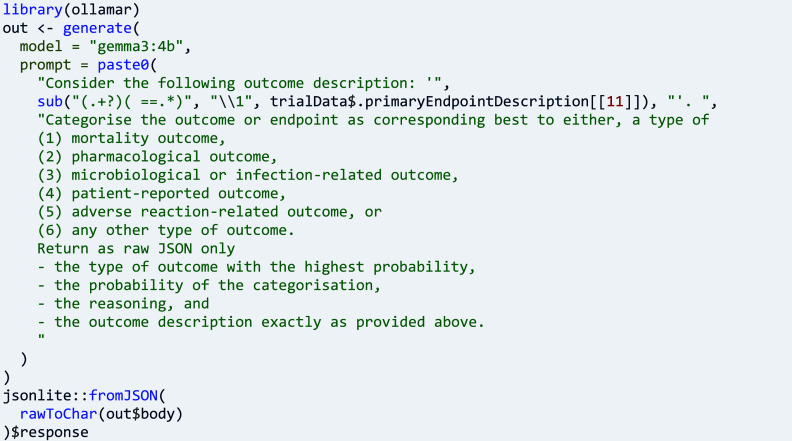

### 3.6. Results-related primary endpoint data

In previous sections, the retrieval from EUCTR did not include result-related data, because this can be quite time-consuming for this register. Results-related data are always retrieved from CTGOV. From CTIS and ISRCTN, there are no results available in a structured format for the foreseeable time.

Retrieving results could have been done already during the first loading of trial data; newly loading trial data that include results overwrites any records of the same trials that were previously loaded (while maintaining user annotations such as those used below):

**Figure:**
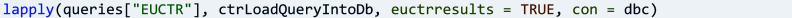

**Figure 3:**
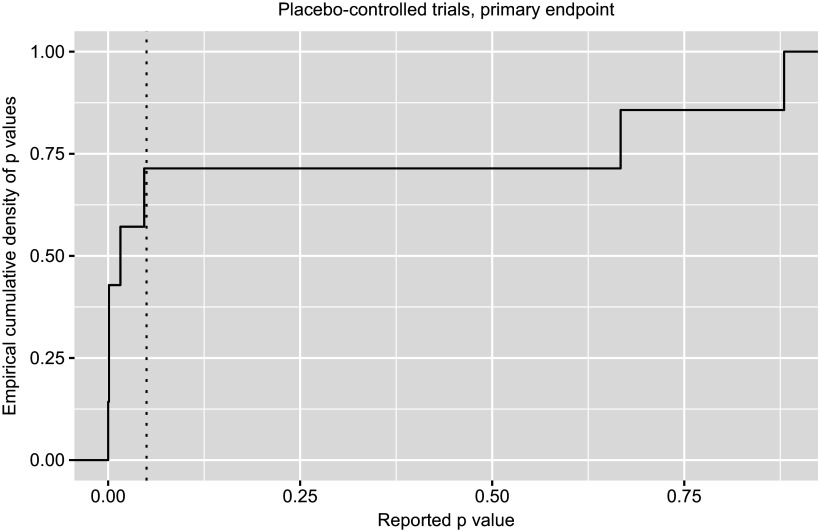
Cumulative density of reported p-values (dotted line, p = 0.05).

Package ctrdata provides data on primary endpoints by analysing various fields in different registers. This simplifies a user’s workflow, for example to explore details of results of null hypothesis significance testing (NHST, Figure 3):

**Figure:**
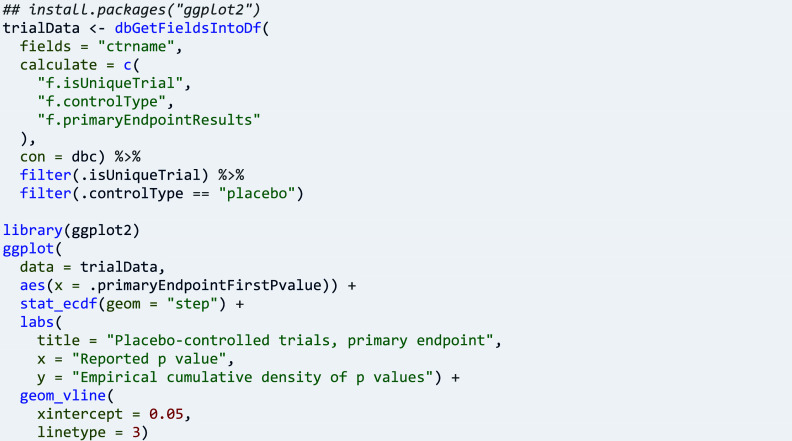

Similarly, statistical methods used for primary endpoint analysis can be tabulated:

**Figure:**
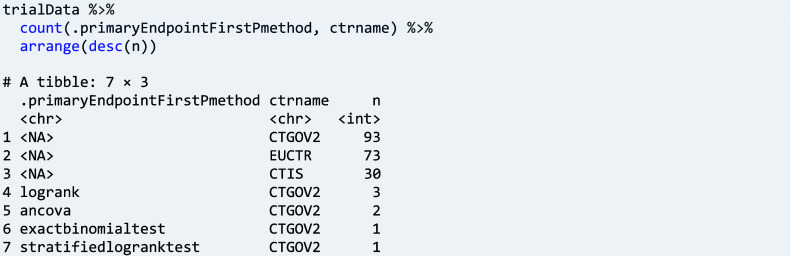

### 3.7. Changes during trial conduct

With the availability of historic versions of registered trials, changes over time can be identified by comparing data of interest across versions. At this time, only CTGOV directly provides historic versions; in addition, ctrdata can create historic versions also for CTIS, when re-running a previous query. Since retrieving historic versions is time-consuming, it has to be specified by the user when calling function ‘ctrLoadQueryIntoDb()’.

For CTGOV, a user has to specify the parameter ‘ctgov2history’ to be either a number (which loads this number of historic versions, at equal time intervals from the first to the current version), a string such as ‘n:m’ (which loads the n^th^ to the m^th^ version) or TRUE (which loads all available versions).

**Figure:**
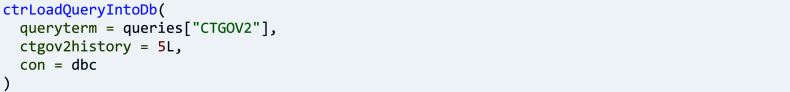

For example, changes in the targeted sample size are an example of the research questions that can be addressed with historic versions:

**Figure:**
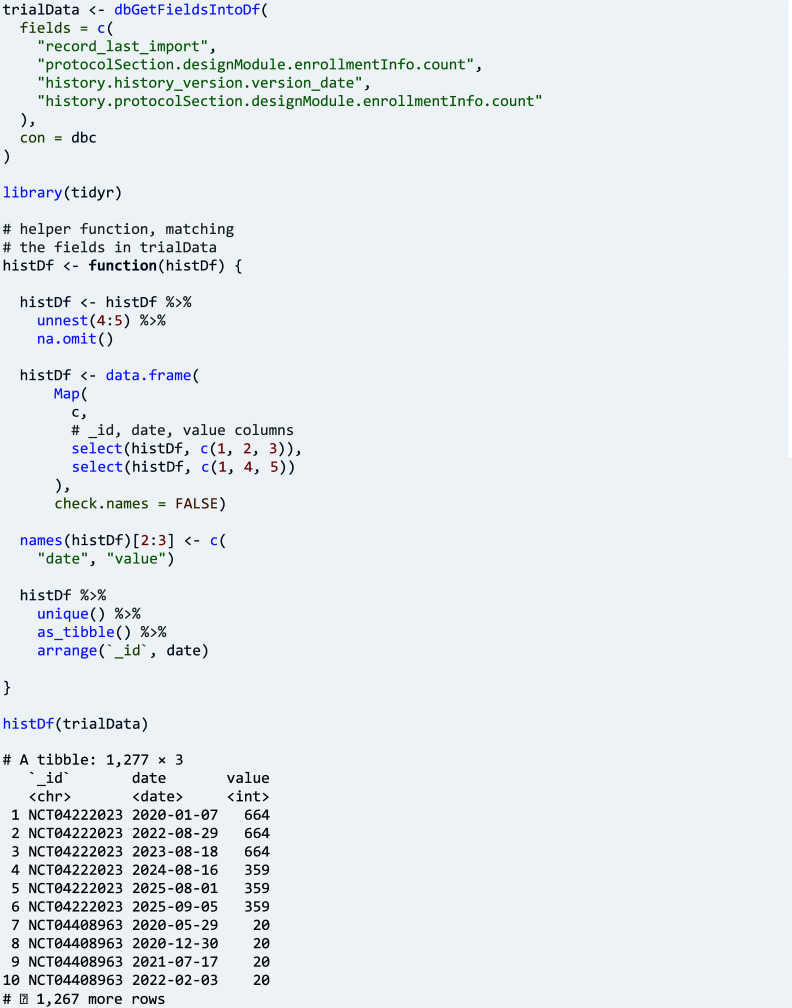

For CTIS, a user has to set parameter ‘ctishistory’ to TRUE and specify ‘querytoupdate’, which moves a current CTIS record in the database into an array of historic versions in the record, before updating the record from CTIS. Thus, historical versions depend on when a user updates a previous CTIS query; for example, changes in recruitment numbers across current and historic versions can be analysed as follows:

**Figure:**
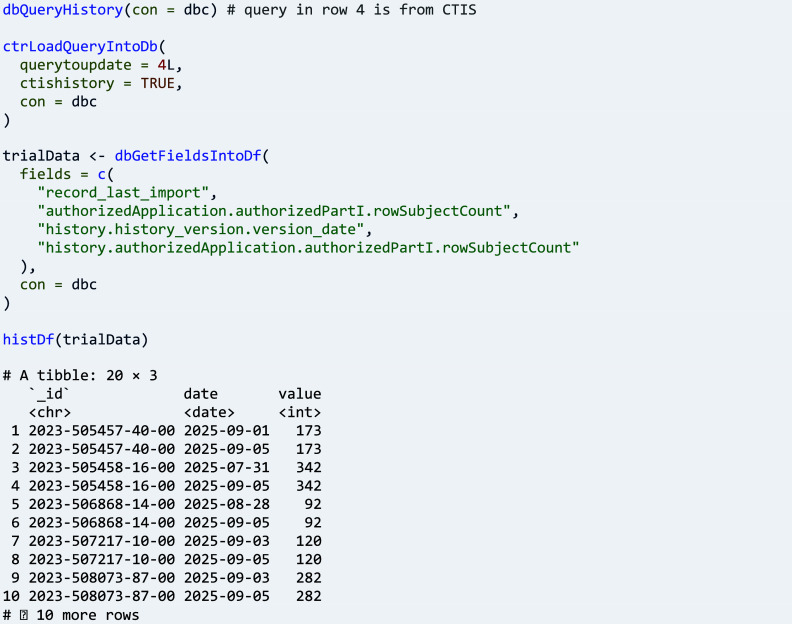

### 3.8. Linking trial and product data

The research activities in clinical trials can lead to data that show the quality, safety, and efficacy of medicines. Research questions about market and patient access to medicines include the progress from trials to product authorisation. They are examples of questions that require two or more data sources for analysis. Here, data on new molecular entities that are authorised medicinal products are retrieved from openFDA^26^:

**Figure:**
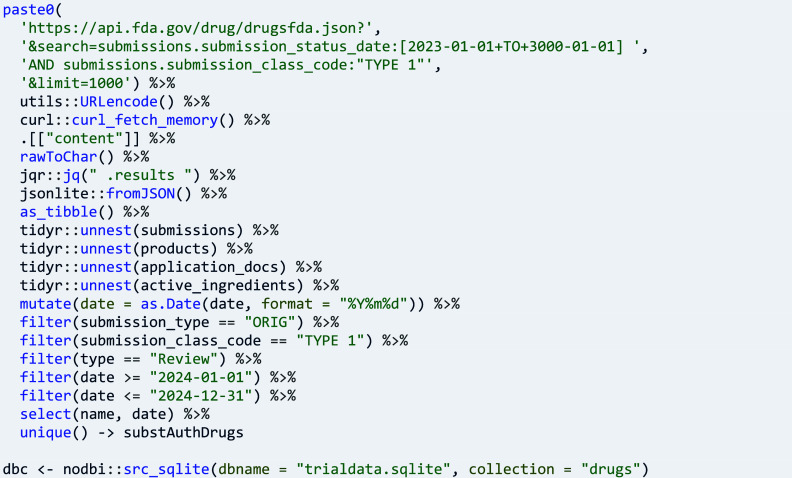

Data on trials are retrieved for the new molecular entities one by one, storing the respective name in the user annotation of the retrieved trials:

**Figure:**
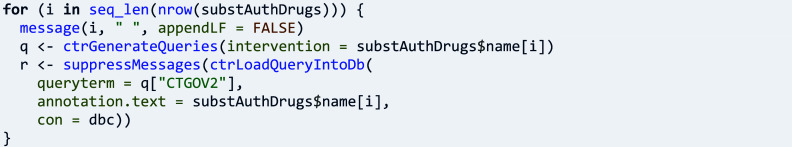

**Figure 4:**
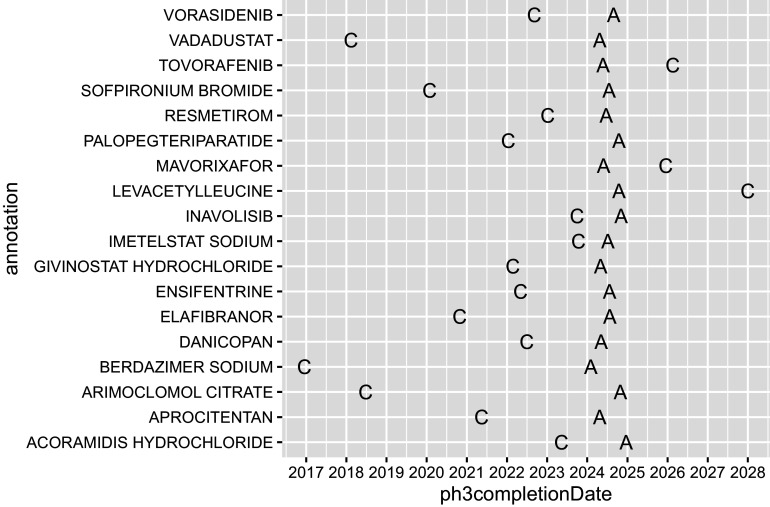
Completion of phase 3 trials and authorisation of medicines.

Therapeutic-confirmatory trials, often referred to as phase 3 trials, are typically needed for regulatory review of applications for marketing authorisation, and here they are merged with product data for a visualisation of trial completion (‘C’) and product authorisation (‘A’, Figure 4):

**Figure:**
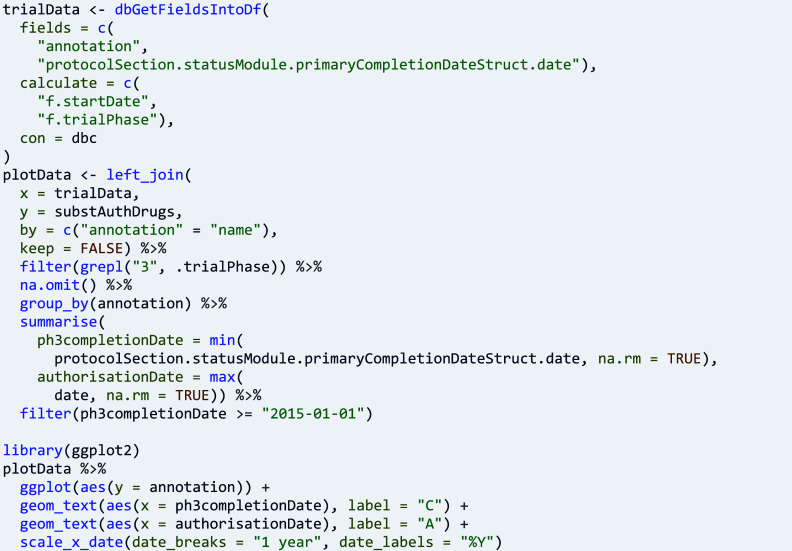

### 3.9. Exploring documents of trials

Study documents offer a rich source of information that is made readily accessible by ctrdata for exploration. Loading documents is activated in function ‘ctrLoadQueryIntoDb()’ by specifying the name of a directory in parameter ‘documents.path’. As a first step for this example, the parameter ‘documents.regexp’ is set to NULL, which causes the function to create empty placeholder files for every document that could be loaded. The names of the files are analysed to obtain an overview of types of available documents.

**Figure:**
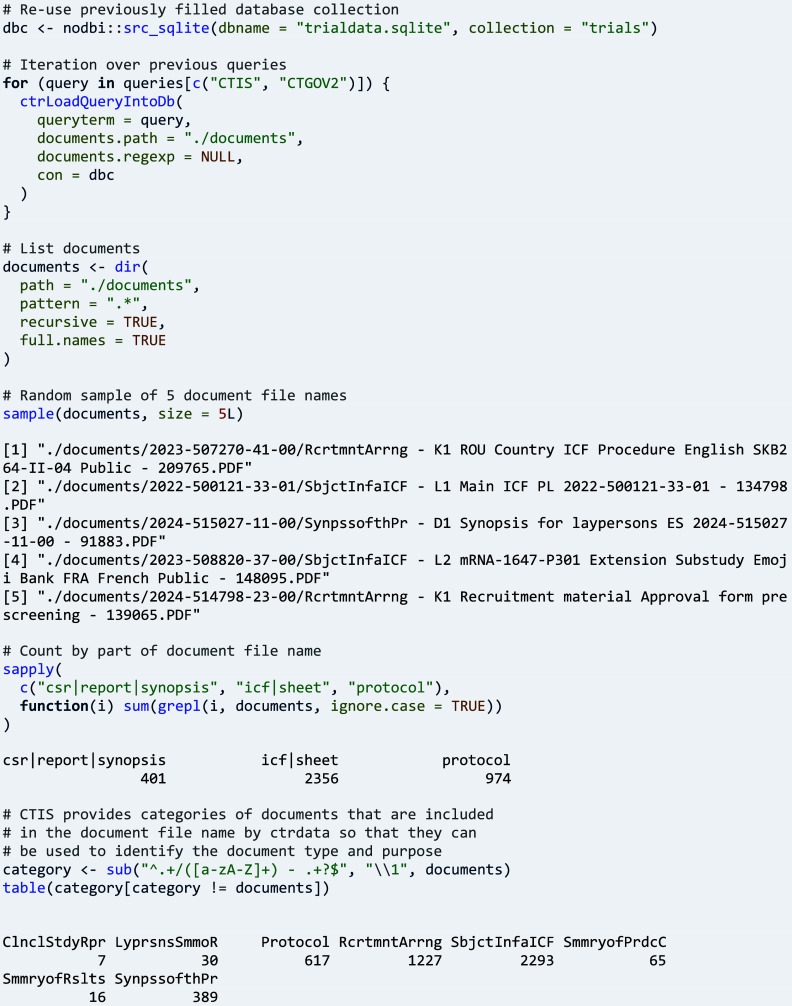

In a second step, parameter ‘documents.regexp’ can be set to a regular expression that causes downloading the files conforming to the expression.

**Figure:**
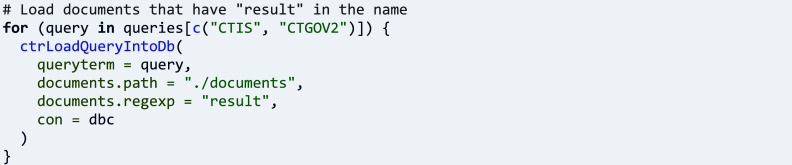

A broad set tools are available for text analysis of document corpora. A recently published tool chain for a retrieval-augmented generation (RAG) workflow in R is ‘ragnar’.^27^ This is used in the following example to search for pharmacokinetics in result documents.

**Figure:**
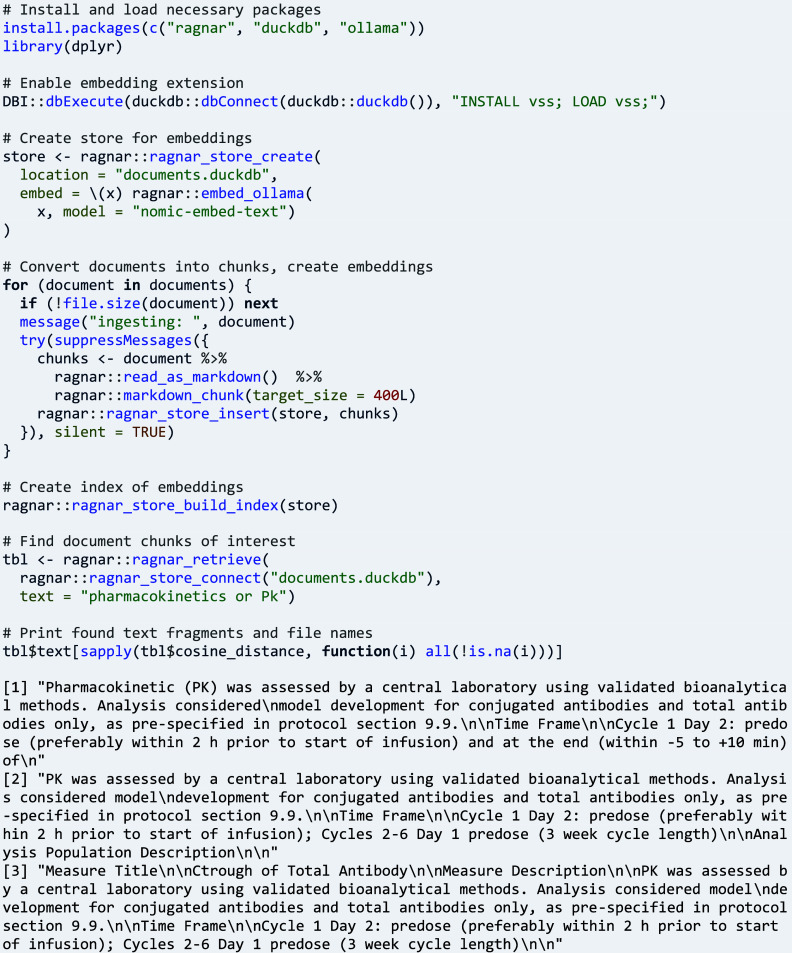

### 3.10. Safety data analysis across registers

Use case 10 is provided in the Supplementary Material to this article.

## 4. Related tools

There is a small number of tools that are more or less related to the objectives and functionality of package ctrdata.

Tools implemented in R include package ‘rclinicaltrials’.^28^ It only supports CTGOV and has functions to download and transform trial data into R objects; it is not available on the Comprehensive R Archive Network (CRAN) and latest commits were pushed to its public repository in 2017. The recent package ‘clintrialx’ only supports CTGOV (or its derivative AACT).^29^ Package ‘cthist’ focuses on historical data from CTGOV.^30^

Other tools include the ‘clinicaltrials-act-trackeR’ which is implemented in Python and uses CTGOV for analysing reporting compliance.^31^ For a comprehensive metadata repository,^32^ tools for downloading and storing data from trial registers CTGOV and EUCTR are implemented in C#.^33^

While package ctrdata covers functionality of the above-mentioned tools, it supports additional and more diverse use cases, as exemplified in this article. Only ctrdata works with four registers, enables to use all registers in the same workflow, and maximally uses all the registers’ public data.

A curated set of R packages relevant for clinical trials, including ctrdata, is in the ‘CRAN Task View: Clinical Trial Design, Monitoring, and Analysis’.^34^

## 5. Limitations and mitigations

Limitations concern the implementation of package ctrdata, the functionality of ctrdata, and the use of registers for research questions.

### 5.1. Implementation

So far, package ctrdata is based on a single developer, and this situation could impact the quality of its implementation and coding, which in turn may impact code comprehensibility and opportunities for involving other developers. This article contributes to the visibility of ctrdata and to attracting contributors. In addition, the following steps mitigate this potential limitation.

For readability and maintainability, code in package ctrdata follows style conventions, uses standard linting, and is well documented with line and function comments as well as a with a comprehensive website that includes vignettes and examples. Over the years, improvement exercises for already functioning code were repeatedly undertaken, including substantial re-implementation, refactoring, or factoring out, and this improved code quality, limited dependencies, stabilised performance, simplified functions, and helped adding registers or adapting to their changes.

Unit and other tests are written at the time that code is written or issues are fixed, and now more than 630 tests cover more than 94% of the code base. A continuous integration pipeline automates testing on several operating systems and with different database backends.

Since 2015, code was regularly committed and pushed to its public repository. Users from across the world contributed with around 50 issues so far, which are visible in the public repository and were resolved typically in hours or days. Since 2016, package ctrdata is made available in the CRAN, which requires stringent checks.

### 5.2. Functionality

Limitations could be seen in the functionality of package ctrdata.

For example, ctrdata does not map or translate related data elements from registers to a single common data model for data storage. Another limitation could be seen in the choice of trial registers that are currently supported by ctrdata. The reasons for these two choices that could be perceived as limitations are discussed in Section 1.

Furthermore, even though no formal approach was used for managing business requirements, the current functionality of ctrdata was informed by a broad variety of questions that users sought to answer with trial register data and by wishes for specific functionalities, such as downloading trial-related documents or providing a correspondence matrix of trial identifiers.

### 5.3. Use for research questions

For research and scientific questions, package ctrdata may be limited if not all relevant registers can be used with ctrdata, or when content of trial registers is incorrect or incomplete. The latter situation can arise from different statutory obligations on trial sponsors. Also, information made publicly available may be ambiguous or not detailed enough for the questions at hand. Early phase trials or trials that were rejected by oversight authorities or ethics committees may have only scarce details or may not be publicly visible at all. Some statutory requirements and information content details are in Table 1.

A challenge with using ctrdata for research questions is that currently interesting topics are often not represented as structured data in trial register information. For example, at this time, platform trials or integrated research platforms^35^ cannot be directly identified in searches or characterised in publicly available data from trial registers. Some registers provide examples for registering trials with such and other less common designs.^36^ Package ctrdata offers a set of trial concepts (Table 3) to mitigate the potential limitations of incomplete controlled vocabularies and of evolving research concepts.

For using CTGOV for research, ten common problems were described.^10^ Conceptually, many of the issues concern any trial register. Package ctrdata can help addressing or mitigating many issues, as exemplified in Table 4.

**Table 4:** Selected issues when using trial registers for research, and how package ctrdata can help

Issue	Opportunities using ctrdata
Registers include studies other than ‘clinical trials’; follow-on studies may have separate records; it is difficult to define a sample of records to answer a specific question; selection bias	ctrdata enables a stepwise selection of records that is documented and reproducible. In addition, ctrdata provides trial concepts for this issue (f.isMedIntervTrial, f.isUniqueTrial; see Table 4). For example, f.trialTitle can be used to filter out extension studies.
Pre-defined data elements in a given register are continually evolving	ctrdata is continually updated to current register contents, and ctrdata also maintains compatibility and code to analyse databases created from earlier versions of register contents (e.g. CTGOV as available until 2024, CTIS before relaunch in 2024)
Type of studies and details of studies change over time; mandatory and optional details of studies change over time; variable extent of quality review	ctrdata makes missing data and varying documentation of details readily visible by enabling tabulations and other analyses and has implemented trial concept functions to handle missing data
Records can be modified any time	ctrdata preserves and documents a snapshot in time of the records by downloading trials into a database and by adding metadata about the queries that were run to add trials to the database (shown with function dbQueryHistory())
Registers do not have or do not make public all information for all studies	ctrdata enables a broader range of research questions to be investigated than other tools or the registers’ website allow to do. In addition, ctrdata enables to rapidly prototype addressing a research question in a simple programmatic approach, which allows the researcher to rapidly appraise the feasibility and decide about a research approach.
Register data can be accessed in several ways; multiple data formats are offered by registers; results data are particularly complex, available often only much later than protocol-related information, may be deferred compared to the primary completion date	ctrdata uses only the most complete format offered by the respective register

## 6. Discussion

This article presents package ctrdata as a new and unique tool that facilitates analysing protocols and results of clinical trials, in detail and for multiple trials from multiple registers at the same time, in an efficient and reproducible approach. The variety of use cases presented above underlines that ctrdata is a simple yet powerful tool. This article also invites collaboration to improve the tool, for which ctrdata is offered in a public repository.

The objective of this package is to support the scientific use of the vast data on clinical trials in public registers, with a view to better leverage existing, accelerate, and improve future research. The tool thus will be of interest for patients and their organisations, clinicians, clinical researchers, pharmaceutical companies, policymakers, health outcome researchers, and medicine regulators. Use cases that cut across interested parties could be envisioned as follows.

Dashboards can be built rapidly on top of package ctrdata using ready-made frameworks, in analogy to those provided during the COVID-19 pandemic for an integrated presentation of global data from different domains (e.g. epidemiology, clinical trials, and molecular biology). Users can adjust and interpret tabulations, aggregations, and visualisations of trial data, including regional availability for potential participation and results of interventions to inform patient-health professional discussions.

Notification systems can employ package ctrdata to query for changes of completed, ongoing, or newly started trials in different registers and link with other health data systems. Users could set themselves up to be automatically notified when news concern medical conditions, types of medicines, trials in their area or otherwise of interest, or when news concern the authorisation and availability of medicines.

Building on ctrdata, patients, researchers, and regulators can better use trial information when writing patient guides, clinical guidelines or assessing a medicine dossier, reviewing methodological details to build experience, and identifying knowledge gaps for which new trials are needed. Scientific works will benefit from the high level of transparency and completeness combined with minimised biases (e.g. availability and spin) where stringent regulatory requirements for trials are applicable. Repeat analyses with reproducible methods can track the performance and change of the clinical research landscape, which will better inform initiatives, policies, and funding.

With respect to technical aspects of ctrdata, the R environment was chosen for reasons including its availability across operating system platforms, its robust infrastructure with package management and quality assurance, and its increasing base of users. To expand the options for exploring global clinical research with package ctrdata, additional registers are considered, but some interesting registers have policies or technical indications against programmatic access and thus will not be included.

For using register data to answer research questions, register curators have published recommendations on how to avoid common problems with trial data. Package ctrdata helps avoid some of these problems, in particular by fully documenting a reproducible search, download, selection, and analysis of trials, which should permit the validation of derived conclusions.

There are interesting challenges for analysing the clinical trial landscape using information from public registers. For example, it remains difficult to track complex clinical trials^37^; here, an approximating concept in package ctrdata may help develop an international approach. It is also sought to address difficulties in identifying and analysing specific trial features such as related to the estimands framework, adaptations of trials during their conduct, and features such as decentralised elements, because these could be impactful for accelerating and improving future trials. Furthermore, data linkage will become a common use case, using trial register information with other public or private data sources, such as exposed by the openFDA APIs^26^ as shown above, publication databases, the EU Open Data Portal^38^ or health outcome, and real-world data bases.^39^ In particular, use cases could link trial register data with regulatory dossiers such as the EU Clinical data publication^40^ or Health Canada’s Public Release of Clinical Information^41^ and, perhaps most importantly, with regulatory reviews of the design, conduct, and results of trials in medicine dossiers as documented in assessment reports.^42^

For the ongoing development of package ctrdata, consideration is given to such challenges as well as to EU initiatives such as for clinical trials^43^ and for collaboration on regulatory science questions.^44^ Questions concerning features of clinical trials are also among the recently updated regulatory science research needs.^45^

In summary, trial registers accumulate data rapidly and continuously: the more the data are explored and used widely, the more they will become valuable information to accelerate clinical research and to improve health care. Package ctrdata enables to make great strides towards these goals.

## Supporting information

Herold supplementary material Herold supplementary material

## Data Availability

The software presented in this paper is available as open source with a permissive licence (MIT) at https://cran.r-project.org/package=ctrdata. This page includes links to the comprehensive documentation website of the package at https://rfhb.github.io/ctrdata/ and to its public repository at https://github.com/rfhb/ctrdata/, which can be used to ask questions, flag issues, and suggest improvements. The programming script for the use cases (Section 3) is provided as Supplementary Material to this publication.

## Supplementary material

To view supplementary material for this article, please visit http://doi.org/10.1017/rsm.2025.10061.
