# Supplementary material for: Aggregating and analysing clinical trials data from multiple public registers using R package ctrdata
Source: Res Synth Methods. 2025 Dec 4;17(3):624–56. doi: 10.1017/rsm.2025.10061 (PMC13126229; doi:10.1017/rsm.2025.10061)
Supplement: Herold supplementary material [file S1759287925100616sup001.zip › 2025-09-06_Ralf_HEROLD_-_ctrdata_Use_case_10_safety_analysis.docx]

# Use case 10

## Safety data analysis across registers

Registers of clinical trials include not only results for endpoints specified for evaluation, but also safety data that is continually collected during the trial. The safety data collection is part of pharmacovigilance activities and employs a highly structured approach to the definition and detection of adverse events, to their assessment including causality and severity, and to the categorisation of the affected organs, functioning or other observations.

In this context, a research question for safety data in clinical trial registers can be to synthesise and analyse events of special interest, for a class of medicinal products. To start, necessary packages are loaded and a database collection is prepared:

## install.packages(c("ctrdata", "RSQLite", "dplyr", "tidyr"))
library(dplyr, warn.conflicts = FALSE)
library(ctrdata)
dbc <- nodbi::src_sqlite(dbname = "trialdata.sqlite", collection = "saes")

This extended use case looks into antibody drug conjugates, which can be associated with specific immunological inflammatory phenomena. Safety data are part of the results-related data in registers such as EUCTR and CTGOV. Trials for such medicinal products, with results and in the therapeutic area oncology can be readily found with queries proposed by ctrdata and loaded from these registers into the database collection:

queries <- ctrGenerateQueries(
 intervention = "antibody drug conjugate",
 onlyWithResults = TRUE,
 condition = "cancer"
)
result <- lapply(
 queries[c("EUCTR", "CTGOV2")],
 ctrLoadQueryIntoDb,
 con = dbc,
 verbose = TRUE,
 euctrresults = TRUE
)

Several hundred records are found and stored:

dbQueryHistory(dbc)

# A tibble: 2 × 4
 `query-timestamp` `query-register` `query-records` `query-term`
 <chr> <chr> <int> <chr>
1 2025-09-04 21:42:35 EUCTR 256 query=cancer AND antibod…
2 2025-09-04 21:42:37 CTGOV2 107 cond=cancer&intr=(antibo…

As a next step in this case, from the retrieved trials, trials of phase 2, 3 or 4 trials have to be selected, which can be assumed to have a sufficiently long observation of the potential occurrences and larger participant numbers. Furthermore, the trials should have an internal control group and not be an extension or follow-up of a previous trial.

This selection is done by calculating corresponding trial concepts that are pre-defined in ctrdata and using them to filter data from all trials. In addition, fields are obtained from the database collection that have been identified (see section 5.2) to hold the safety data of interest:

trialData <- dbGetFieldsIntoDf(
 fields = c(
 "adverseEvents.reportingGroups", # EUCTR
 "adverseEvents.seriousAdverseEvents.seriousAdverseEvent", # EUCTR
 "resultsSection.adverseEventsModule.seriousEvents", # CTGOV2
 "resultsSection.adverseEventsModule.eventGroups" # CTGOV2
 ),
 calculate = c(
 "f.isUniqueTrial",
 "f.trialPhase",
 "f.trialTitle",
 "f.controlType"
 ),
 con = dbc
) %>%
 filter(.isUniqueTrial) %>%
 filter(!grepl("extension", .trialTitle)) %>%
 filter(grepl("2|3|4", .trialPhase)) %>%
 filter(.controlType %in% c("placebo", "active"))

The overall approach is to obtain details of allocation or reporting groups, to merge this with details (counts) of all recorded types of serious adverse events per group, and finally to merge EUCTR and CTGOV data into a single data frame.

The safety data are represented in a nested data structure, in a hierarchy of several levels. To simplify handling such data, ctrdata provides the function “dfTrials2Long()”, which generates for each scalar data item (variable) a single row in a long and narrow data frame. This is a unique function that penetrates each level of the data structure and returns the name of the variable (in JSON dot-notation) and its value. From the input data frame with around 26 rows, the function returns a new data frame with around 38.500 rows, in which around 40 variables are repeated, e.g. within combinations of allocation groups, periods and outcomes, for which a unique identifier is created per trial:

trialDataLong <- trialData %>%
 dfTrials2Long()

trialDataLong %>%
 slice_sample(n = 5L)

# A tibble: 5 × 4
 `_id` identifier name value
 <chr> <chr> <chr> <chr>
1 2011-000033-36-BE 25.5 adverseEvents.seriousAdverseEvents.serious… 0
2 NCT03544281 19.21 resultsSection.adverseEventsModule.serious… EG020
3 NCT01997333 29.2 resultsSection.adverseEventsModule.serious… EG001
4 NCT03734029 26.1 resultsSection.adverseEventsModule.serious… 2
5 NCT02980341 42.6 resultsSection.adverseEventsModule.serious… EG005

First, EUCTR data are used to obtain the reporting groups details:

library(tidyr)

# Obtain reporting groups for adverse events
# with total number of subjects: with an AE,
# with an SAE and exposed, for each group
euctrGroups <- dfName2Value(
 df = trialDataLong,
 valuename = paste0(
 "adverseEvents.reportingGroups.reportingGroup.",
 "(id|title|subject)")
) %>%
 na.omit() %>%
 pivot_wider(id_cols = c(`_id`, identifier)) %>%
 select(-identifier) %>%
 rename(
 title =
 adverseEvents.reportingGroups.reportingGroup.title,
 reportingGroupId =
 adverseEvents.reportingGroups.reportingGroup.id)

Next, the serious adverse events (SAEs) details are obtained. The code shows the usefulness of the long data frame from dfTrials2Long, from which a range of fields can be readily extracted and pivoted into a wide format that has one row for each SAE for each group for each trial:

# Obtain details for SAEs, e.g. organ system,
# dictionary, occurrences and fatalities
euctrSaesDetails <- dfName2Value(
 df = trialDataLong,
 valuename = paste0(
 "adverseEvents.seriousAdverseEvents.seriousAdverseEvent.",
 "(.*)")
)

# For each trial and each AR,
# - first create a set without the values (counts)
# - then create a set of only the values (counts)
# - merge into table in which one row corresponds
# to one reporting group for one AR for one trial
euctrSaesDetails <- left_join(
 euctrSaesDetails %>%
 pivot_wider(id_cols = c(`_id`, identifier)) %>%
 filter(!grepl("[.]", identifier)) %>%
 select(-contains("values")),
 euctrSaesDetails %>%
 filter(grepl("[.]", identifier)) %>%
 pivot_wider(id_cols = c(`_id`, identifier)) %>%
 mutate(identifier = sub("([0-9]+)[.]?.*", "\\1", identifier)),
 by = c("_id", "identifier")
) %>%
 # Shorten reportingGroupId
 rename(
 reportingGroupId =
 adverseEvents.seriousAdverseEvents.seriousAdverseEvent.values.value.reportingGroupId) %>%
 # Remove empty reportingGroupId
 filter(!is.na(reportingGroupId)) %>%
 # Remove column created by dfTrials2Long, but not
 # needed anymore as next merge uses reportingGroupId
 select(-identifier)

To complete the EUCTR steps, the group and SAE details are merged:

saesEuctr <- left_join(
 x = euctrSaesDetails,
 y = euctrGroups,
 by = c("_id", "reportingGroupId")
) %>%
 # Simplify variable names, removing leading parts
 rename_with(~ sub(
 "adverseEvents.seriousAdverseEvents.seriousAdverseEvent.(|values.value.)", "", .x),
 starts_with("adverse")
 ) %>%
 # Select variables
 select(
 `_id`,
 title,
 term,
 subjectsExposed,
 occurrences
 ) %>%
 # Convert to integer, because dfTrials2Long
 # extracted nested data as character strings
 mutate(
 subjectsExposed = as.integer(subjectsExposed),
 occurrences = as.integer(occurrences)
 )

saesEuctr %>%
 slice_sample(n = 5L)

# A tibble: 5 × 5
 `_id` title term subjectsExposed occurrences
 <chr> <chr> <chr> <int> <int>
1 2011-000509-29-BE Trastuzumab Emtansine Smal… 403 1
2 2018-001321-68-3RD Mylotarg with Standard C… Acut… 183 0
3 2012-004879-38-BE T-DM1 + P Thro… 223 1
4 2018-001321-68-3RD Mylotarg: Treatment for … Vomi… 9 0
5 2012-000660-22-BE Trastuzumab Emtansine 3.… Hypo… 69 1

Second, CTGOV data are mangled, starting with details on reporting groups:

ctgovGroups <- trialData %>%
 select(`_id`, resultsSection.adverseEventsModule.eventGroups) %>%
 unnest(resultsSection.adverseEventsModule.eventGroups) %>%
 rename(groupId = id) %>%
 filter(!is.na(groupId))

The SAE details are obtained from a different field, and the stats field provides counts by allocation group, type of SAE and organ system.

ctgovSaesDetails <- trialData %>%
 unnest(resultsSection.adverseEventsModule.seriousEvents) %>%
 unnest(stats)

To complete the CTGOV steps, the group and SAE details are merged:

saesCtgov <- left_join(
 x = ctgovSaesDetails,
 y = ctgovGroups,
 by = c("_id", "groupId")
) %>%
 select(
 `_id`,
 title,
 term,
 numAtRisk,
 numAffected
 )

Finally, the data from EUCTR and CTGOV (after adjusting column names) can directly be appended to each other. This provides the sought synthesis data set of serious adverse events of special interest for a specific class of medicines, which can be further interrogated, for example with respect to dosing or other data with which the trials can be linked:

bind_rows(
 saesEuctr,
 saesCtgov %>%
 # names as in saesEuctr
 rename(
 subjectsExposed = numAtRisk,
 occurrences = numAffected
 )
) %>%
 slice_sample(n = 10L)

# A tibble: 10 × 5
 `_id` title term subjectsExposed occurrences
 <chr> <chr> <chr> <int> <int>
 1 NCT02988817 Dose-escalation Part: Enapotam… Card… 10 0
 2 NCT04209855 Mirvetuximab Soravtansine COMP… 227 1
 3 NCT02001623 Dose Escalation Part: 0.6 mg/kg Muco… 3 0
 4 NCT02606305 Mirvetuximab Soravtansine 5 mg… Pulm… 4 0
 5 NCT03149549 Parts A & A2 10 mg/kgQ3W (5 in… Hypo… 8 0
 6 NCT02573324 Depatuxizumab Mafodotin, Radia… STAP… 273 0
 7 NCT03248492 Part 1: DS-8201a High Dose Dysp… 21 1
 8 NCT03544281 Main Study Phase: Belantamab M… Dehy… 12 0
 9 NCT03544281 Main Study Phase: Belantamab M… Anxi… 4 0
10 NCT03525678 Main Study: GSK2857916 3.4 mg/… Basa… 99 1
